# Studying the API Distribution of Controlled Release Formulations Produced via Continuous Twin-Screw Wet Granulation: Influence of Matrix Former, Filler and Process Parameters

**DOI:** 10.3390/pharmaceutics16030341

**Published:** 2024-02-28

**Authors:** Phaedra Denduyver, Chris Vervaet, Valérie Vanhoorne

**Affiliations:** Laboratory of Pharmaceutical Technology, Department of Pharmaceutics, Ghent University, Ottergemsesteenweg 460, B-9000 Ghent, Belgium; phaedra.denduyver@ugent.be (P.D.); chris.vervaet@ugent.be (C.V.)

**Keywords:** continuous manufacturing, twin-screw granulation, wet granulation, controlled release, hydroxypropyl methylcellulose, process variables, formulation variables, content uniformity

## Abstract

Hydroxypropyl methylcellulose (HPMC) is a preferred hydrophilic matrix former for controlled release formulations produced through continuous twin-screw wet granulation. However, a non-homogeneous API distribution over sieve fractions with underdosing in the fines fraction (<150 µm) was previously reported. This could result in content uniformity issues during downstream processing. Therefore, the current study investigated the root cause of the non-homogeneous theophylline distribution. The effect of process parameters (L/S-ratio and screw configuration) and formulation parameters (matrix former and filler type) on content uniformity was studied. Next, the influence of the formulation parameters on tableting and dissolution behavior was investigated. Altering the L/S-ratio or using a more aggressive screw configuration did not result in a homogeneous API distribution over the granule sieve fractions. Using microcrystalline cellulose (MCC) as filler improved the API distribution due to its similar behavior as HPMC. As excluding HPMC or including a hydrophobic matrix former (Kollidon SR) yielded granules with a homogeneous API distribution, HPMC was identified as the root cause of the non-homogeneous API distribution. This was linked to its fast hydration and swelling (irrespective of the HPMC grade) upon addition of the granulation liquid.

## 1. Introduction

Controlled release matrix tablets are of high interest to improve patient adherence, minimize fluctuations in drug plasma levels, avoid adverse drug effects and reduce health care costs [1]. Hydroxypropyl methylcellulose (HPMC) is a well-established hydrophilic polymer to formulate controlled release matrix tablets. It is a water-soluble, non-ionic and non-toxic cellulose ether that is enzyme resistant, stable over pH range 3–11 and classified as GRAS by Food and Drug Administration (FDA) [2,3]. Various HPMC grades are available which differ in substitution type (ratio of hydroxypropoxy and methoxy groups) and molecular weight, determining the solubility and gelling rate.

Many studies focused on the processing of HPMC-based controlled release formulations via batch-wise wet granulation techniques, namely high shear and fluidized-bed granulation [4,5,6,7,8,9]. Despite the advantages of continuous manufacturing (improved cost-efficiency, easier scale-up, lower environmental footprint, enhanced flexibility) and the emerging of twin-screw wet granulation (TSWG), the production of HPMC matrix tablets through this continuous technique remains poorly studied [10,11,12,13]. A study of Thompson and O’Donnell [14] processed the controlled release (CR) excipients, HPMC and Kollidon SR, via TSWG. However, this yielded thin noodle-like shape granules due to a ‘rolling’ phenomenon during the granulation process. Altering the screw design was the most effective means to produce near-spherical particles. However, the impact on CR properties of the granules was not studied and no dissolution experiments were performed. Vanhoorne et al. [15] reported the formation of twisted elongated granules only at excessive liquid-to-solid ratio (L/S-ratio) for the same formulation. However, by excluding microcrystalline cellulose (MCC) as filler from the formulation, the shape of the HPMC granules was similar to those of immediate release formulations. In addition, the influence of filler and process parameters (screw speed, throughput, barrel temperature and screw design) on the granule and tablet properties of an HPMC-based controlled release formulation was studied. In a follow-up study, the influence of the HPMC grade and particle size of theophylline on the critical quality attributes of the granules and tablets was investigated [16]. This showed a non-homogeneous distribution of theophylline over the different sieve fractions with underdosing in the fines fraction (<150 µm), but this could not be correlated to the properties of the HPMC grades.

Vanhoorne et al. [16] reported how to obtain CR granules with similar shape to immediate release formulations, however could not explain the inhomogeneous API distribution over the different sieve fractions. As a homogeneous distribution of the active pharmaceutical ingredient (API) is essential to avoid content uniformity issues during downstream processing (fluidized-bed drying and tableting) and to adhere to the quality-by-design principles, this study investigates the root cause of the non-homogeneous API distribution in CR granules after processing via TSWG. The influence of process (L/S-ratio and screw configuration) and formulation parameters (filler and matrix former type) on the API distribution over the different granule sieve fractions was studied. In addition, the influence of formulation parameters on tableting and dissolution behavior was investigated.

## 2. Materials and Methods

### 2.1. Materials

Theophylline was used as slightly soluble model drug (BCS class I) (Siegfried, Zofingen, Switzerland) [17]. Two hydrophilic HPMC matrix formers (90SH-4000SR and 65SH-4000SR) were kindly donated by ShinEtsu (Niigata, Japan). According to the Ph. Eur., the substitution types of HPMC 90SH and HPMC 65SH are 2208 (19.0–24.0% methoxy and 4.0–12.0% hydroxypropoxy) and 2906 (27.0–30.0% methoxy and 4.0–7.5% hydroxypropoxy) respectively [18]. The hydrophobic matrix former Kollidon^®^ SR was kindly donated by BASF (Ludwigshafen, Germany). Kollidon^®^ SR is a mixture of 80% polyvinyl acetate and 19% povidone (Kollidon^®^ 30). α-Lactose monohydrate (Pharmatose^®^ 200M, DFE Pharma, Goch, Germany), mannitol (Parteck^®^ M200, Merck, Darmstadt, Germany), dicalcium phosphate (DCP) (Emcompress^®^ Anhydrous Powder, JRS Pharma, Rosenberg, Germany) and MCC (Avicel^®^ PH101, FMC Health and Nutrition, Cork, Ireland) were used as fillers. Pharmatose 200M, Parteck M200 and Emcompress Anhydrous Powder were kindly donated. Magnesium stearate (Ligamed MF-2-V, Peter Greven, Bad Münstereifel, Germany) was used as lubricant during tableting. The particle size distribution (PSD) of the API and fillers are listed in Table 1.

### 2.2. Preparation of Granules

**Figure 1:**
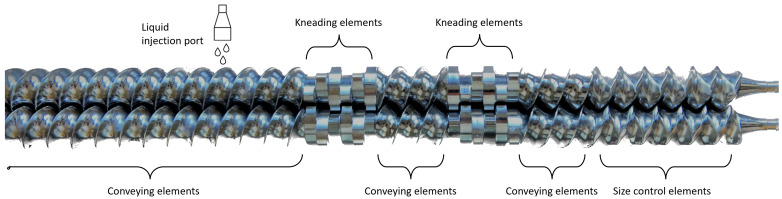
Screw configuration 1 with material flow from left to right. Kneading zones consists of 6 kneading elements (length-to-diameter ratio 1/4) in a stagger angle of 60°. In screw configuration 2 and 3, one and two kneading elements were placed in angle of 90°, respectively. Granulation liquid was added just before first kneading zone.

Theophylline (20% *w*/*w*), matrix former (0 or 20% *w*/*w*) and filler (60 or 80% *w*/*w*) were preblended in a 20 L tumbling mixer (Inversina Bioengineering, Wald, Switzerland) at 25 rotations per minute (rpm) for 15 min. An overview of the formulations, as well as their abbreviations used throughout the manuscript, are shown in Table 2. Granulation experiments were performed on the twin-screw granulator of the continuous ConsiGma^TM^-25 system (GEA Pharma Systems, Wommelgem, Belgium). The preblends were transferred to the loss-in-weight feeder (KT20, K-Tron Soder, Niederlenz, Switzerland), and subsequently fed into the granulation unit at a throughput of 20 kg/h. The granulation unit consists of two co-rotating screws with a length-to-diameter ratio of 20/1. The screws consist of conveying elements, two kneading zones with each six kneading elements (KE), separated by conveying elements, and size control elements at the end (Figure 1). Three different screw configurations (SCs) were used. In SC 1, the KE were all placed in a forward stagger angle of 60°. In SC2 and SC3, one and two KE were placed in a forward stagger angle of 90° respectively, while keeping the other KE in a forward stagger angle of 60°. Screw speed was kept constant at 700 rpm.

Demineralized water as granulation liquid was pumped into the granulator barrel just before the first kneading zone using two silicon tubings (1.6 mm internal diameter) connected with two 1.6 mm nozzles and two out-of-phase peristaltic pumps (Watson Marlow, Cornwall, UK). A range of L/S ratios (varying in steps of 0.01) was preliminarily examined to define the L/S ratios used when manufacturing a larger amount of granules, i.e., a low L/S ratio yielding <15% fines and a high L/S ratio yielding <5% fines (Table 2). The temperature during granulation was kept constant at 30 °C by an active cooling system of the granulator barrel. The torque was monitored by a built-in torque-gauge at 1-s intervals. After stabilization of the torque, 1000 g granules were collected and subsequently tray-dried in an oven at 40 °C till a loss-on-drying value between 1 and 3% was obtained. Moisture content of 5 g granules was determined using a moisture analyzer (HC 103, Mettler-Toledo, Zaventem, Belgium) at 105 °C until the weight was constant for 30 s.

### 2.3. Preparation of Tablets

The granules produced with fillers DCP and MCC at highest L/S-ratio and using SC1 were milled through a 1000 µm grater screen at 900 rpm using the Quadro comil (U10, Quadro, ON, Canada) integrated in the ConsiGma^TM^-25 line. The milled granules were blended with 0.5% magnesium stearate in a tumbling blender for 5 min at 49 rpm (T2F, W. A. Bachofen, Basel, Switzerland). Tablets were prepared on a compaction simulator (STYL’One Evolution, Medelpharm, Beynost, France) equipped with one pair of flat-faced Euro B punches of 10 mm diameter (Natoli Engineering Company, Saint Charles, MO, USA). Compression tests were carried out at main compaction pressures of 64, 127, 191, 255, 318, 382 and 446 MPa with a targeted tablet weight of 500 mg. Based on the tabletability plots, tablets compressed at 318 MPa were selected for dissolution testing.

### 2.4. Particle Size Distribution

The PSD of the excipients was measured in triplicate by laser diffraction (Malvern Mastersizer 3000, Malvern Instruments, Worcestershire, UK). The measurements were performed via the dry dispersion method in volumetric distribution mode. Prior to the measurement, a pressure tritation was performed to determine the pressure required to measure the primary particle size of the excipients, i.e., a pressure which disperses agglomerates into primary particles without shattering of the primary particles. The dry dispersion unit was set at a specific feed rate to ensure an obscuration ranging between 0.5 and 8%. The 10%, 50% and 90% cumulative undersize fraction of the volume distribution was reported as Dv_10, Dv_50 and Dv_90.

### 2.5. Evaluation of Granules

#### 2.5.1. API Content Uniformity

To determine the distribution of API, granules were sieved in seven fractions using a Retsch VE 1000 sieve shaker (Haan, Germany). First, a sample of 100 g granules was obtained using a rotary cone sample divider (Laborette 27, Fritsch Idar-Oberstein, Germany). Then, the sample of granules was placed on a series of sieves (150, 250, 500, 710, 1000 and 1400 µm) during 5 min at an amplitude of 1.5 mm. The amount of granules retained on each sieve was determined and isolated. A calibration curve was set-up by measuring the absorbance of different theophylline solutions (0.001 to 0.020 mg/mL) at a wavelength of 272 nm through UV spectroscopy (UV-1650PC, Shimatzu Benelux, Antwerp, Belgium). The isolated sieve fractions were dissolved in demineralized water (10 mg/mL), diluted 200 times, and their absorbance was measured. The theophylline content in these samples was calculated based on the calibration curve (R^2^ of 0.9992) and corrected for the theophylline content and loss-on-drying value of the pre-blend.

#### 2.5.2. Friability Analysis

The granule friability was determined in triplicate using a friabilator (PTF 300 Pharma Test, Hainburg, Germany). Before the test, the granule fraction <250 µm was removed. A representative sample of 10 g granules (I_wt_) with 200 glass beads (mean diameter 4 mm) was placed in a drum rotating at a speed of 25 rpm during 10 min, subjecting the granules to falling shocks. Afterwards, the glass beads were removed and the mass retained on a 250 µm sieve (F_wt_) was determined. The friability was calculated as $[\left(I_{wt}-F_{wt}\right)/I_{wt}]\times 100$.

#### 2.5.3. Particle Size Analysis

The granule size distributions and aspect ratio were determined via dynamic image analysis using the QICPIC^TM^ system (Sympatec, Clausthal-Zellerfeld, Germany), equipped with a vibrating feeder system (Vibri/L^TM^) for gravimetric feeding of the granules. Prior to measurement, a sample of granules was obtained using a rotary cone sample divider (Laborette 27, Fritsch Idar-Oberstein, Germany). Measurements were performed in triplicate. The median granule size (d_50_) (defined as the diameter of a circle of equal projection area) was calculated using WINDOX 5 software (Sympatec, Clausthal-Zellerfeld, Germany). The percentage of fines, yield and oversized were defined as the fraction <150 µm, 150–1500 µm and >1500 µm, respectively. The median aspect ratio (a_50_) of the granules was calculated by the WINDOX 5 software to evaluate the shape of the granules.

### 2.6. Evaluation of Tablets

#### 2.6.1. Tensile Strength Analysis

The hardness, thickness, and diameter of the tablets (*n* = 10) was determined using a hardness tester (ST50, Sotax, Saint-Louis, France). The tensile strength of the tablets was calculated according to Equation (1) described by Fell and Newton [19].

(1) $$TS=\frac{2F}{\pi dt}$$

where *F*, *d* and *t* denote the diametral crushing force, tablet diameter and tablet thickness, respectively.

#### 2.6.2. Dissolution Testing

Dissolution tests were performed (*n* = 3) in 900 mL demineralized water using the United States Pharmacopeia (USP) method with paddles (708-DS, Agilent, Santa Clara, CA, USA). The temperature of the dissolution medium was maintained at 37.0 ± 0.5 °C, while the rotation speed was set at 100 rpm. Samples of 5 mL were withdrawn and filtered with a 35 µm porous filter (FIL035-PT, Pharmatest, Hainburg, Germany) after 0.5, 1, 2, 4, 6, 8, 12, 16, 20 and 24 h. The absorbance of the samples was measured at a wavelength of 272 nm using a UV spectrophotometer (UV-1650PC, Shimadzu Benelux, Antwerp, Belgium). The similarity between the dissolution profiles of the two HPMC grades was assessed by calculating the similarity factor $f_{2}$ through Equation (2) as described by Shah et al. [20]. $R_{t}$ and $S_{t}$ represent the cumulative drug release at sample point *t* of the tablets containing HPMC 2208 and 2906 and *n* equals the number of total sample points. Maximum one sample point with a cumulative drug release higher than 85% may be included to avoid bias in the similarity assessment, therefore the number was set at 9 and 10 respectively for the MCC and DCP formulations containing both HPMC grades. Dissolution profiles are considered similar if $f_{2}$ is ≥50, which corresponds to an average difference of less than 10% at all sample time points [20].

(2) $$f_{2}=50log_{10}\left\{{\left[1+\frac{1}{n}\sum_{t=1}^{n}{\left(S_{t}-R_{t}\right)}^{2}\right]}^{-1/2}x100\right\}$$

## 3. Results and Discussion

### 3.1. Evaluation of Granulation Process

**Figure 2:**
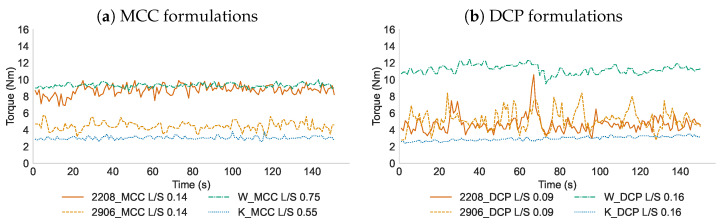
Torque profiles with matrix formers HPMC type 2208, HPMC type 2906, Kollidon SR and without matrix former. While a common L/S-ratio was used for HPMC-based formulations, the L/S-ratio was adapted for formulations containing Kollidon SR and without matrix former to avoid processing issues.

The effect of matrix former type on the torque values of the granulation process was evaluated. As the swelling behavior of the matrix formers was of interest, the torque values at similar L/S-ratios for HPMC matrix formers were compared (i.e., 0.09 and 0.14 for formulations containing DCP and MCC, respectively), even though this resulted in a different granule quality. For the MCC formulations containing Kollidon SR or without matrix former, the low L/S-ratio was chosen: 0.55 and 0.75, respectively. For the DCP formulations, a common L/S-ratio (i.e., 0.16) was selected for the formulations without matrix former and with Kollidon SR. No processability issues were experienced as the torque values were all below the maximum torque (20 Nm) (Figure 2). The torque using HPMC substitution type 2208 (8.8 ± 0.6 Nm) was higher compared to HPMC type 2906 (4.5 ± 0.5 Nm) for the MCC formulation (Figure 2a). In contrast, similar torque values were obtained using HPMC type 2208 (4.7 ± 1.0 Nm) or HPMC type 2906 (5.4 ± 1.3 Nm) for the DCP formulation (Figure 2b). HPMC type 2208 exerts faster swelling behaviour than HPMC type 2906 due to its higher fraction of hydroxypropyl groups, resulting in a higher viscosity upon wetting and requiring more rotational forces (i.e., torque) to rotate the screws [21]. The lower torque values of the high density DCP formulation compared to the MCC-based formulation can be attributed to the lower filling degree of the granulator barrel, minimizing the impact of swelling behavior of the HPMC grades upon contact with water [22]. Without matrix former, relatively high torque values were obtained for both formulations (9.3 ± 0.2 Nm and 8.7 ± 0.6 Nm, for MCC and DCP respectively). Although no viscous and/or swelling materials were included in these formulations which could be responsible for the frictional resistance to flow, the high amount of liquid needed to form high-quality granules of formulations containing 20% theophylline and 80% water-insoluble filler resulted in the high torque values. During continuous granulation at the same L/S-ratio of formulations containing the water-soluble fillers lactose and mannitol but without the inclusion of HPMC, lower torque values (2.4 ± 0.1 Nm and 1.4 ± 0.1 Nm, respectively) were obtained compared to formulations with HPMC type 2208 (10.0 ± 0.6 Nm and 7.6 ± 0.6 Nm, respectively). This torque study indicated that the inclusion of the hydrophilic matrix former HPMC type 2208 caused a significant increase in resistance to flow due to its fast gelling rate upon hydration. In contrast, the matrix former Kollidon SR yielded the lowest torque values for both formulations as it induced limited friction at a given screw speed due to its hydrophobic nature.

### 3.2. Influence of L/S-Ratio, Screw Configuration and Filler Solubility on API Homogeneity of Granules Containing HPMC Type 2208

**Figure 3:**
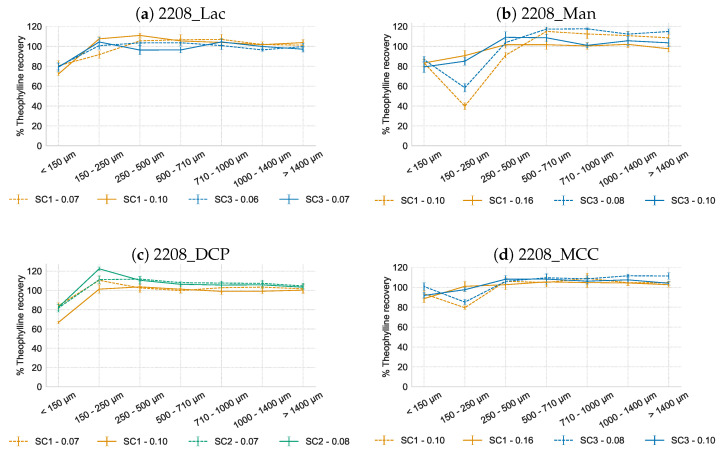
Theophylline recovery (% of theoretical content) in function of granule sieve fraction, when processed at low (dashed line) and high (full line) L/S-ratio using SC1 (orange), SC2 (green) or SC3 (blue).

Figure 3 shows a non-homogeneous API distribution over the sieve fractions irrespectively of the L/S-ratio, SC or filler type. Typically theophylline underdosing was observed in the smaller size fractions (i.e., fines < 150 µm, and also the 150–250 µm fraction for the MCC and mannitol-based formulations), and compensated by a limited API overdosing in the larger sieve fractions. For lactose and DCP (Figure 3a,c), a higher L/S-ratio had a negligible effect on the API distribution over the different sieve fractions, independent of the screw configuration. In contrast for mannitol and MCC (Figure 3b,d), modifying the screw configuration (i.e., SC2 and SC3) improved the API distribution at high L/S-ratio. At low L/S-ratio, a strong underdosing was detected in the 150–250 µm fraction and limited underdosing in the <150 µm fraction, whereas at high L/S-ratio only limited underdosing in both fractions was seen. As this effect of L/S-ratio on the API distribution over the different sieve fractions could not be attributed to the solubility of the fillers, other properties of the filler particles which affect the wetting behavior (e.g., contact angle, surface energy, particle size, porosity) will contribute to this effect [23]. The poor wetting of the material and subsequent hindered liquid distribution at low L/S-ratio could prevent part of the material from participating in the granulation process, resulting in a non-homogeneous API distribution over the sieve fractions. MCC has a high water binding capacity, which could hinder the liquid distribution [24]. The higher primary particle size of mannitol, Dv_50 of 135.3 µm, compared to a Dv_50 of 46.1, 14.5 and 69.9 µm of lactose, DCP and MCC, respectively (Table 1), might contribute to the poor wetting. It is hypothesized that the larger mannitol particles used in current experiments—despite being water-soluble and having a higher intrinsic dissolution rate compared to lactose—dissolve slower due to their smaller surface area [25], also given the short residence time of the material in the granulation barrel during TWSG. Subsequently, those particles are unable to form nuclei during the granulation process and remain ungranulated even after experiencing shear in the kneading zones. The effect of excipient PSD on API distribution should be examined in future research.

When including MCC in the formulation as a filler, it resulted in the lowest API underdosing (88.6% ± 4.1) of the fines fraction when processed at the high L/S ratio, vs. 72.2% ± 1.5, 83.3% ± 3.4 and 66.7% ± 1.2, for lactose, mannitol and DCP, respectively. MCC has a high water binding capacity due to its porous structure and therefore experiences some degree of swelling in contact with water [24]. As both HPMC and MCC exhibit this behavior, this could favor the homogeneous API distribution over the different sieve fractions. In a previous study of our research group, elongated granules were obtained when processing HPMC and other cellulose derivates at high L/S-ratios [15]. In the current study, similar aspect ratios were obtained for all fillers processed at high L/S-ratio: 0.65, 0.65, 0.60 and 0.63 for lactose, mannitol, DCP and MCC, respectively. No issues on particle shape were experienced as these aspect ratios of the CR granules were close to those reported for immediate release formulations (typically between 0.64 and 0.70) [15].

Introducing more shear during granulation by increasing the stagger angle of the kneading elements (i.e., granulation using SC2 and SC3) had no effect on the API distribution. For the DCP formulation, granules were produced on SC2 instead of SC3 due to processability issues. Van Den Dries et al. [26] found a relationship between granule breakage and granule homogeneity during high-shear granulation whereby a dynamic situation of granule breakage is associated with the continuous exchange of particles resulting in homogeneous granules. Although the higher shear generated by SC2 and SC3 compared to SC1 increased the rate process of breakage, the gel layer of the viscous HPMC granules appears to prevent this breakage mechanism [4,27]. Therefore, no difference in API homogeneity between the different SCs was observed.

### 3.3. Influence of Matrix Former Types on API Homogeneity of CR Granules

#### 3.3.1. Without Matrix Former

**Figure 4:**
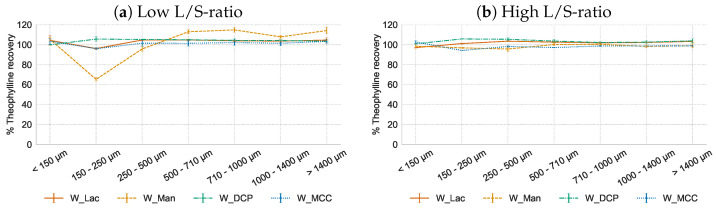
Formulations without matrix former (20% *w*/*w* theophylline and 80% *w*/*w* filler). Figures show theophylline recovery (% of theoretical content) as a function of the granule sieve fractions at low and high L/S-ratio, respectively.

In Figure 4, the theophylline recovery in function of sieve fractions of formulations without matrix former is shown. A homogeneous API distribution was obtained if HPMC type 2208 was not included, except for mannitol at low L/S-ratio. These results demonstrated that HPMC type 2208 influenced the growth mechanism of the individual particles, resulting in a non-uniform API distribution. Without HPMC, granule breakage and continuous exchange of particles can occur whereby granules with a homogeneous API distribution are obtained [26]. The API underdosing in the 150–250 µm fraction at low L/S-ratio for the mannitol formulation disappeared at a higher liquid content. This effect was also noticed when including HPMC type 2208 and mannitol (Figure 3b), and could be attributed to the larger particle size of mannitol (Section 3.2 and Table 1). Although no binder is present to facilitate the bonds in these formulations, at high L/S-ratio high-quality granules were obtained with a friability below 20% and less than 7% fines.

#### 3.3.2. Hydrophilic Matrix Formers HPMC Type 2906 and 2208

**Figure 5:**
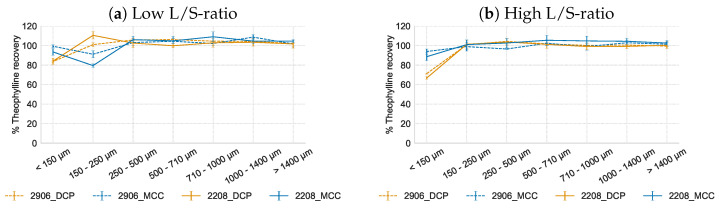
Theophylline recovery (% of theoretical content) in function of the sieve fraction for formulations with HPMC type 2906 and 2208 as matrix former at low and high L/S-ratio.

The influence of two HPMC grades on the API distribution in function of the sieve fractions was studied (Figure 5). At low L/S-ratio, HPMC type 2906 yielded a slightly more homogeneous API distribution than HPMC type 2208, but theophylline underdosing in the <150 µm (DCP) or 150–250 µm (MCC) fractions was still observed. The difference in underdosing between DCP and MCC is similar as described in Section 3.2. At lower L/S ratios, due to the limited amount of water, the initial swelling rate of the HPMC particles plays a bigger role. Therefore, HPMC type 2906, which contains less hydrophilic groups and which consequently swells slower than type 2208, yielded slightly better results. The slower swelling of HPMC type 2906 in combination with MCC was also reflected in the torque results (Section 3.1). At high L/S-ratio, a similar content uniformity was obtained for both HPMC grades as they can fully exert their swelling behavior when sufficient liquid is available.

#### 3.3.3. Hydrophobic Matrix Former Kollidon SR

**Figure 6:**
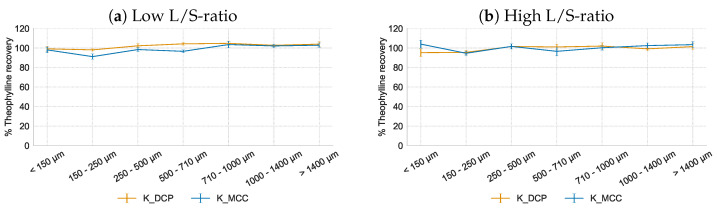
Theophylline recovery (% of theoretical content) in function of granule sieve fraction for formulations with Kollidon SR as matrix former at low and high L/S-ratio.

Kollidon SR as matrix former yielded a homogeneous theophylline distribution over the granule sieve fractions for both fillers and L/S-ratios (Figure 6). This confirmed the previous results which indicated that the non-homogeneous API distribution over the different sieve fractions is caused by HPMC due to its rapid swelling upon hydration during wet granulation which limits granule breakage and continuous exchange of particles. As Kollidon SR does not swell in contact with water, it did not affect the API distribution over the granule size fractions. These results emphasised the importance of the behaviour of the matrix former upon its initial contact with the granulation liquid as this can trigger and maintain heterogeneous distribution of API in the granules. High aspect ratios were obtained for the granules with Kollidon SR (0.70 and 0.75 for DCP and MCC, respectively) and without matrix former (0.75, 0.73, 0.73 and 0.73 for lactose, mannitol, DCP and MCC, respectively). In contrast, the HPMC-based formulations resulted in granules with an aspect ratio varying between 0.60 and 0.65, indicating that these granules were more elongated. This difference in granule shape is also linked to the swelling of HPMC upon hydration as the thick gel layer hinders the consolidation of the granules into a more spherical shape [4].

### 3.4. Influence of Formulation Variables on Tablet Quality and Dissolution Profile

**Figure 7:**
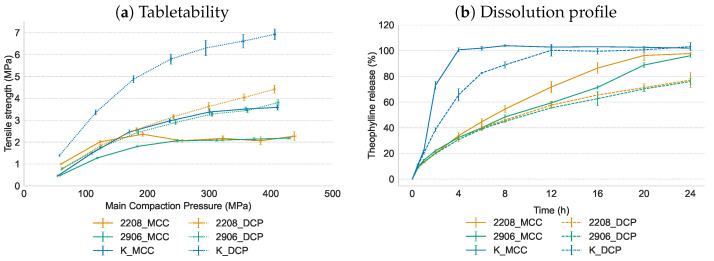
Tabletability and dissolution profile of tablets produced with theophylline as drug, HPMC type 2208, HPMC type 2906 or Kollidon SR as matrix former and DCP or MCC as filler. Tablets for dissolution were compressed at 318 MPa.

Tablets were produced using granules which contain MCC or DCP in combination with the different matrix formers and which were produced at the high L/S-ratio. For both HPMC grades, strong tablets (>1.7 MPa) were obtained at a main compaction pressure of 191 MPa (Figure 7a). No significant differences were seen in compaction behaviour of the HPMC grades, which is in alignment with previous results from Vanhoorne et al. [16]. Although MCC is an excipient with excellent compressibility and compactibility, tablets with a lower tensile strength compared to DCP were obtained [28,29] since wet granulation reduces the compactibility of MCC due to a decrease in porosity of the primary MCC particles [30,31]. There is less energy used for plastic deformation at the particle interfaces and more energy for fragmentation during compaction of MCC granules [32]. As shown in Figure 7b, a controlled release profile without a burst effect was obtained for both HPMC matrix formers. No significant differences in dissolution profile were seen between the HPMC grades as the similarity factor $f_{2}$ was 56.1 and 89.3 respectively for the MCC and DCP formulations containing the two HPMC grades. After 24 h, complete drug release was achieved for MCC-based tablets, whereas DCP-based tablets released only 76% of the drug content. Bendgude et al. [33] studied the effect of excipients on swelling and erosion of compressed HPMC tablets and reported that DCP had no noticeable effect on the swelling, erosion and hydration of HPMC K15M. In contrast, MCC swells upon hydration resulting in disintegration properties, influencing the swelling and erosion behavior of HPMC and tablet disintegration by wicking and swelling. Although wet granulation causes loss of some of its disintegration properties [34], faster drug release of MCC-based tablets was attributed to the disintegrating properties of MCC.

The formulations containing Kollidon SR as matrix former yielded strong tablets (Figure 7a), especially in combination with DCP as filler. Kollidon SR exhibits excellent compaction properties as it deforms plastically during tableting [35]. Although Kollidon SR yielded granules with a homogeneous API distribution and these granules could be processed into strong tablets, this matrix former showed no controlled release (Figure 7b). Whereas HPMC-based tablets form a viscous barrier upon hydration to reduce the drug release rate, Kollidon SR forms a matrix (due to its insoluble polyvinyl acetate fraction) whereby its polyvinyl pyrrolidone fraction leaches from the hydrophobic matrix to create addition pores. While the current Kollidon SR formulation failed to provide controlled drug release optimization of the formulation (e.g., increasing the matrix former concentration or combination of different matrix formers) could improve the sustained release capacity of the granules.

## 4. Conclusions

When processing controlled release formulations via continuous wet granulation HPMC caused a non-homogeneous API distribution over the different granule sieve fractions due to its rapid swelling upon contact with the aqueous granulation liquid preventing granule breakage and subsequently the exchange of particles during granule growth. Addition of more granulation liquid yielded granules with a slightly better theophylline distribution when using mannitol and MCC as filler, but still resulted in underdosing of the fines fraction (<150 µm). Although a difference in API concentration over the sieve fractions was observed between fillers, this was not linked to differences in their solubility. While the addition of MCC as filler improved the API distribution, the more pronounced API underdosing in granules containing mannitol as filler which were processed at low L/S-ratio might be linked to the larger primary particle size of the filler. The effect of excipient PSD on API homogeneity should be further investigated in future research. Strong tablets with a controlled release profile were obtained using both HPMC grades in combination with MCC or DCP as filler. Kollidon SR as matrix former yielded granules with a homogeneous content uniformity but did not allow to control the API release from tablets in this formulation.

## Figures and Tables

**Table 1:** Overview of the particle size distribution of API and fillers.

	Theophylline	Pharmatose 200M	Parteck M200	Emcompress Anhydrous Powder	Avicel PH101
Dv_10 (µm)	7.91 ± 0.02	7.58 ± 0.08	59.07 ± 0.88	1.57 ± 0.03	26.07 ± 0.12
Dv_50 (µm)	30.90 ± 0.08	46.13 ± 0.05	135.33 ± 2.62	14.53 ± 0.33	69.93 ± 0.09
Dv_90 (µm)	88.97 ± 0.21	119.33 ± 0.47	337.33 ± 9.53	32.90 ± 0.22	147.33 ± 0.47

**Table 2:** Overview of the composition of the granulated formulations with their process parameters.

Formulation ^(a)^	Theophylline (%)	Matrix Former (%)			Filler (%)				L/S-Ratio (Low–High)		
		**HPMC** **90SH-** **4000SR**	**HPMC** **65SH-** **4000SR**	**Kollidon** **SR**	**Pharmatose** **200M**	**Parteck** **M200**	**Emcompress** **Anhydrous** **Powder**	**Avicel** **PH101**	**SC1** **12KE60°**	**SC2** **10KE60°** **2KE90°**	**SC3** **8KE60°** **4KE90°**
2208_Lac	20	20	-	-	60	-	-	-	0.07–0.10	-	0.06–0.07
2208_Man	20	20	-	-	-	60	-	-	0.10–0.16	-	0.08–0.10
2208_DCP	20	20	-	-	-	-	60	-	0.07–0.10	0.07–0.08	NA
2208_MCC	20	20	-	-	-	-	-	60	0.10–0.16	-	0.08–0.10
W_Lac	20	-	-	-	80	-	-	-	0.10–0.14	-	-
W_Man	20	-	-	-	-	80	-	-	0.14–0.20	-	-
W_DCP	20	-	-	-	-	-	80	-	0.10–0.16	-	-
W_MCC	20	-	-	-	-	-	-	80	0.75–0.90	-	-
2906_DCP	20	-	20	-	-	-	60	-	0.09–0.13	-	-
2906_MCC	20	-	20	-	-	-	-	60	0.14–0.20	-	-
K_DCP	20	-	-	20	-	-	60	-	0.14–0.16	-	-
K_MCC	20	-	-	20	-	-	-	60	0.55–0.85	-	-

^(a)^ First index for matrix former type _ Second index for filler type. 2208 = HPMC 90SH-4000SR; W = without matrix former; 2906 = HPMC 65SH-4000SR; K = Kollidon SR//Lac = lactose; Man = mannitol; DCP = dicalcium phosphate; MCC = microcrystalline cellulose. In SC1, the KE in kneading zones were all placed in a forward stagger angle of 60° and in SC2 and SC3, one and two KE were placed in a forward stagger angle of 90°, respectively.

## Data Availability

Data is contained within the article.
